# Mechanical failure of plate breakage after open reduction and plate fixation of displaced midshaft clavicle fracture – a possible new risk factor: a case report

**DOI:** 10.1186/s13256-019-2046-3

**Published:** 2019-04-28

**Authors:** Ron Batash, Ronen Debi, Dan Grinberg, Maayan Shema, Avi Elbaz, Yeshaiau Benedict

**Affiliations:** 1 Department of Orthopedic Surgery, Barzilai Medical Center, 2 Hahistadrut Street, 78278 Ashkelon, Israel; 2 AposTherapy Research Group, Herzliya, Israel

**Keywords:** Clavicle, Open reduction and plate fixation, Failure, Plate breakage, Case report

## Abstract

**Background:**

Plate breakage is one form of construct failure after a clavicle fracture treated with an open reduction and plate fixation. A recent study evaluated construct failure after an open reduction and plate fixation and reported a construct failure rate of 6.9% of which 1.9% were related to broken plates. Plate breakage is rare, thus, there are insufficient data regarding risk factors, pathogenesis, or how to avoid it.

**Case presentation:**

This case report presents an unusual case of a 35-year-old Caucasian man, 7 weeks after open reduction and internal plate fixation of a fracture in the middle third of his clavicle, who developed breakage of the implant. Surgery was advised, the implant was retrieved, the fracture was reduced, and a new bridging locking plate was implanted.

**Conclusions:**

In the current case it seems that the use of a bridging plate, the fundamental anatomical structure of the clavicle and the forces that are applied on it, the lack of discipline in complying with the postoperative functional restrictions, and an unclear “patient expectation” process were the main reasons for the failure. These aspects should be carefully considered and addressed in clavicle fractures.

## Background

Clavicle fractures are a common traumatic injury with a reported incidence of between 5 and 10% of all fractures and approximately 44% of injuries to the shoulder girdle [1]. Approximately 70–80% of these fractures are located in the middle third of the bone and are displaced [2]. Traditionally, the conservative approach was the most common choice, especially in fractures of the medial or lateral end of the clavicle, if fracture fragments remained stable [3]. However, in severely displaced fractures the clinical evidence on conservative treatment suggests poor outcomes such as malunion and nonunion [4]. In the last decade, there has been growing evidence that supports the use of operative treatments, mainly due to fewer nonunion rates, better functional outcomes, earlier resumption of daily activities, prompt pain relief, and restoration of anatomic clavicular shape [5, 6]. Definite and possible indications for operative treatment include open fracture, skin tenting, neurovascular compromise, substantial displacement, comminution, and shortening (> 1 to 2 cm) [2]. The implants mostly used can be divided into two groups: intramedullary devices as nails and extramedullary devices as plates; plates can be subdivided into reconstruction plates and small fragment locking compression plates [7].

Although high success rates of plate fixation have been shown, complications have also been reported. The latter include implant failure, infections, implant prominence, poor cosmesis, nonunion, and refracture after removal of the plate [8]. Implant failure such as breakage, mechanical failure, irritation, and angulation occurs in between 6.3 and 8.5% of the cases [9–11]. A recent study evaluated construct failure after an open reduction and plate fixation and reported a construct failure rate of 6.9% of which 1.9% were related to broken plates [7]. The only significant risk factor they found was that all the plates were used to bridge the fracture as opposed to neutralizing or compressing it. Plate breakage is rare, thus, there are insufficient data regarding risk factors, pathogenesis, or how to avoid it. This case report presents an unusual case of a patient with a midshaft clavicle fracture following a plate breakage with the aim of providing more information on this complication and recommends a working plan that might help to prevent these cases in advance.

## Case presentation

**Fig. 1:**
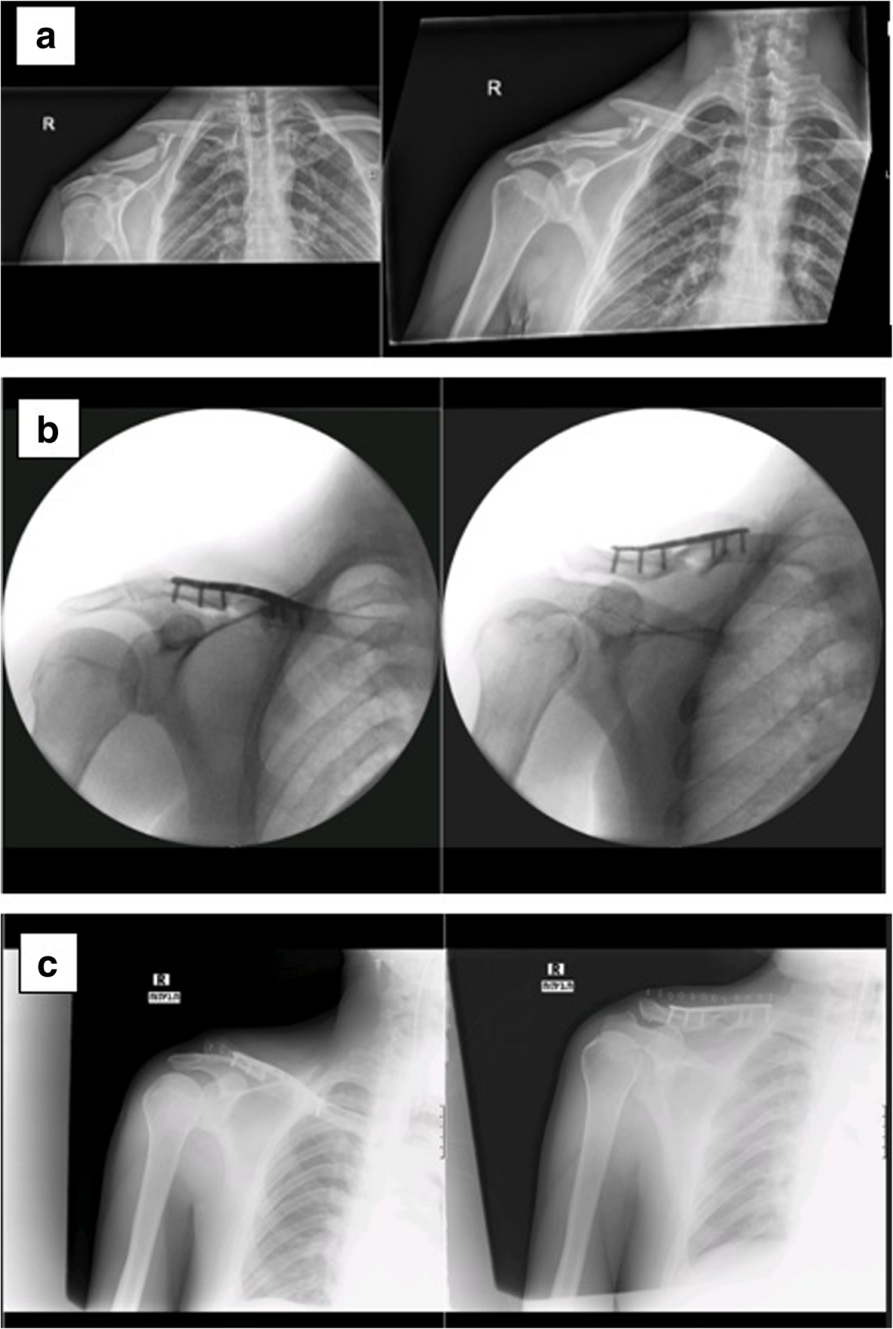
**a** X-ray: displaced comminuted right midshaft clavicle fracture; **b** intraoperative imaging; **c** postoperative X-ray with reduction and bridging osteosynthesis with an anatomical contoured locked plate

**Fig. 2:**
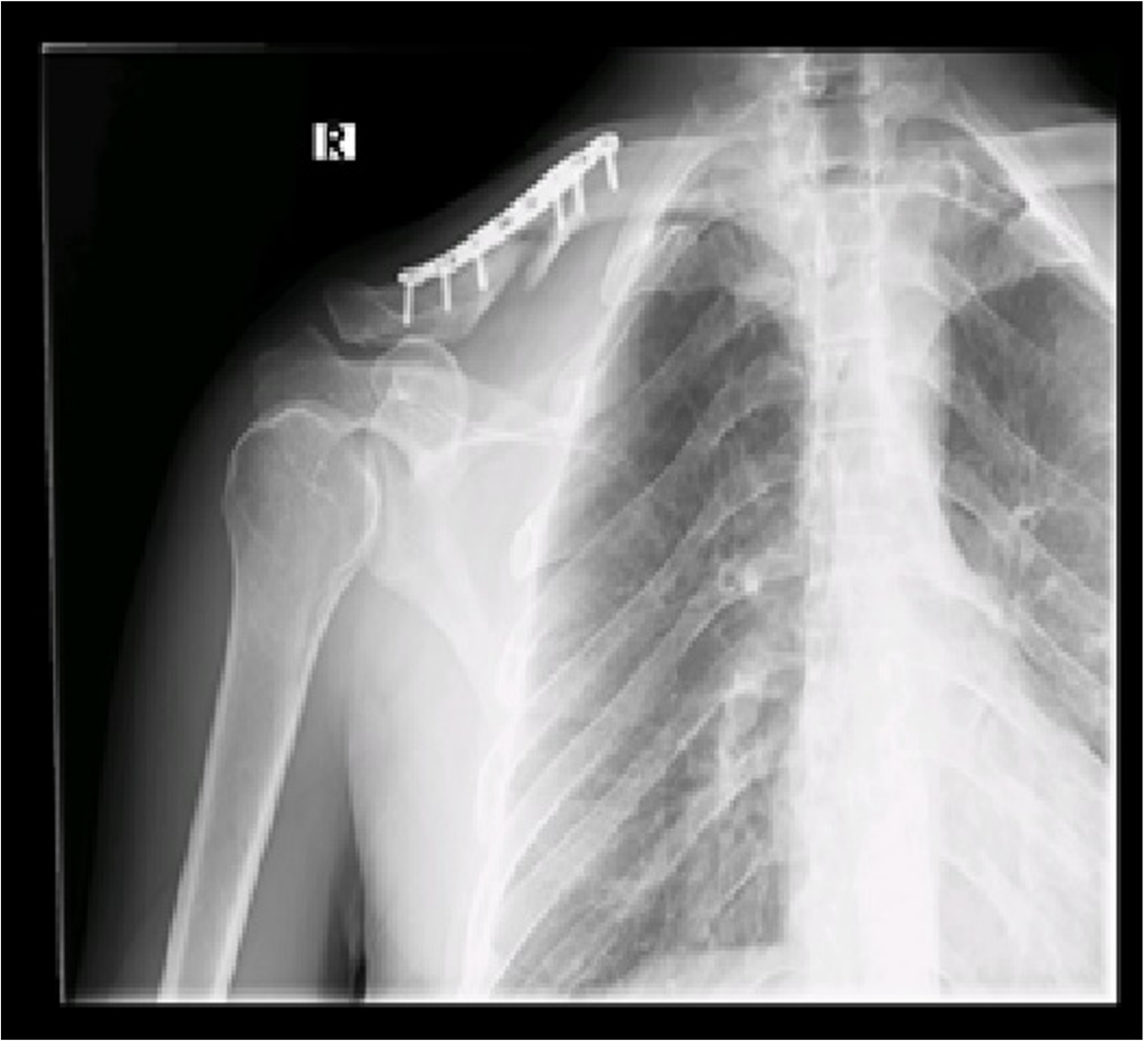
Postoperative 2-week follow-up X-ray

A 35-year-old Caucasian man fell laterally on his right shoulder due to a hoverboard accident. On X-ray at our emergency room (ER), a displaced comminuted right middle third clavicle fracture, with clavicle shortening was diagnosed (Fig. 1a). He was otherwise healthy with no routine medications or allergies. He is right-handed; his occupation is car electrician and he wished to regain his hand function in order to get back to work as soon as possible. Considering his age, level of physical activity, fracture pattern, and his expectations, surgery was advised. The operation was performed 10 days later. A superior approach to his clavicle using right-sided Acumed Locking Clavicle Plate was applied. Intraoperative and postoperative imaging were performed (Fig. 1b, c). After the operation he was treated with analgesia, his shoulder was immobilized in a sling, and physical therapy was recommended with restricted range of motion of < 80° abduction. He was asked to return to a standard follow-up examination after 2 weeks, in which a standard X-ray demonstrated the fracture fixated by the locking plate (Fig. 2). He reported feeling good and was released with the recommendation of continuing physical therapy while avoiding lifting heavy weights.

**Fig. 3:**
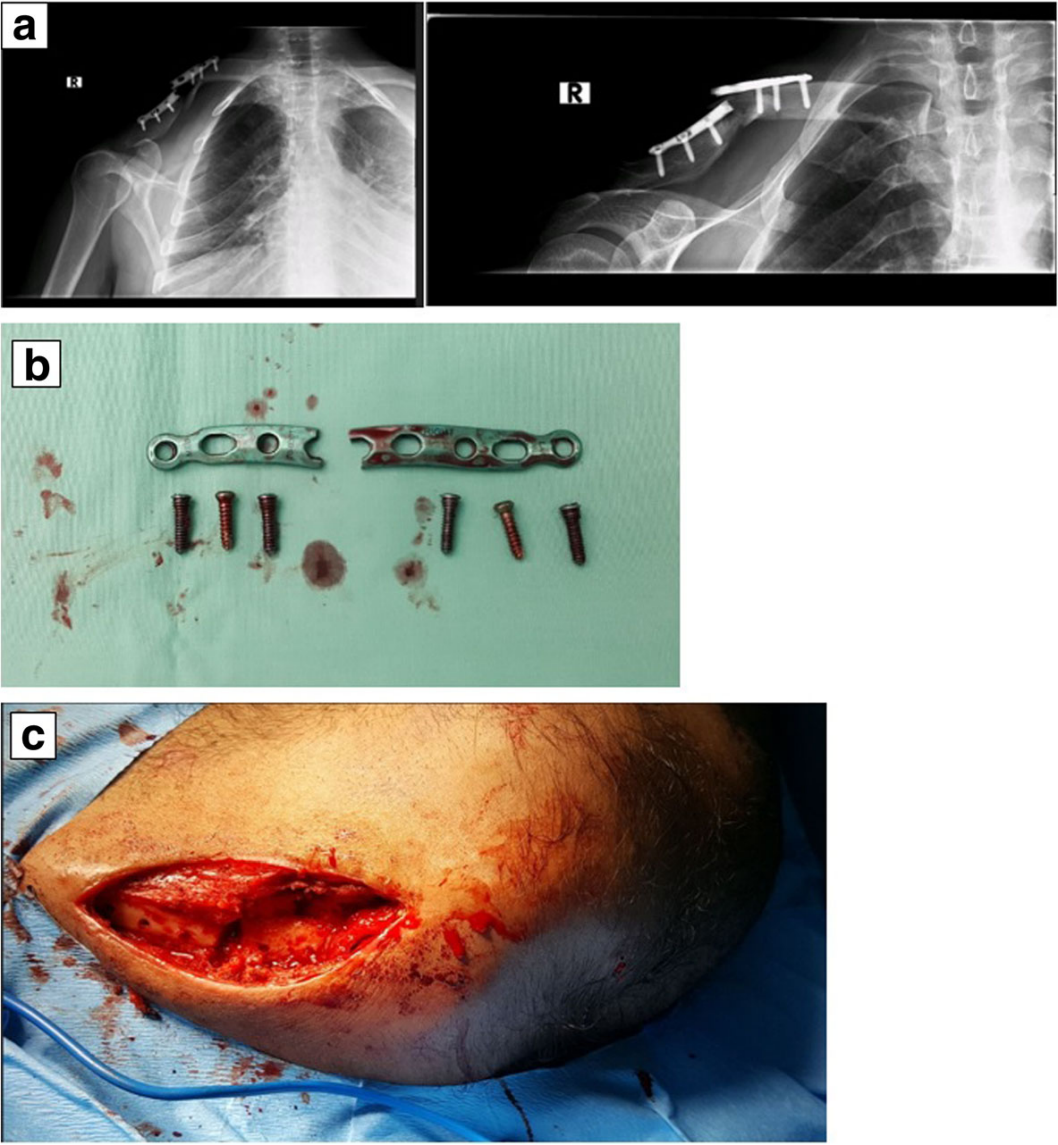
**a** X-ray: implant failure with plate breakage; **b** plate breakage; **c** an image of the failure area

**Fig. 4:**
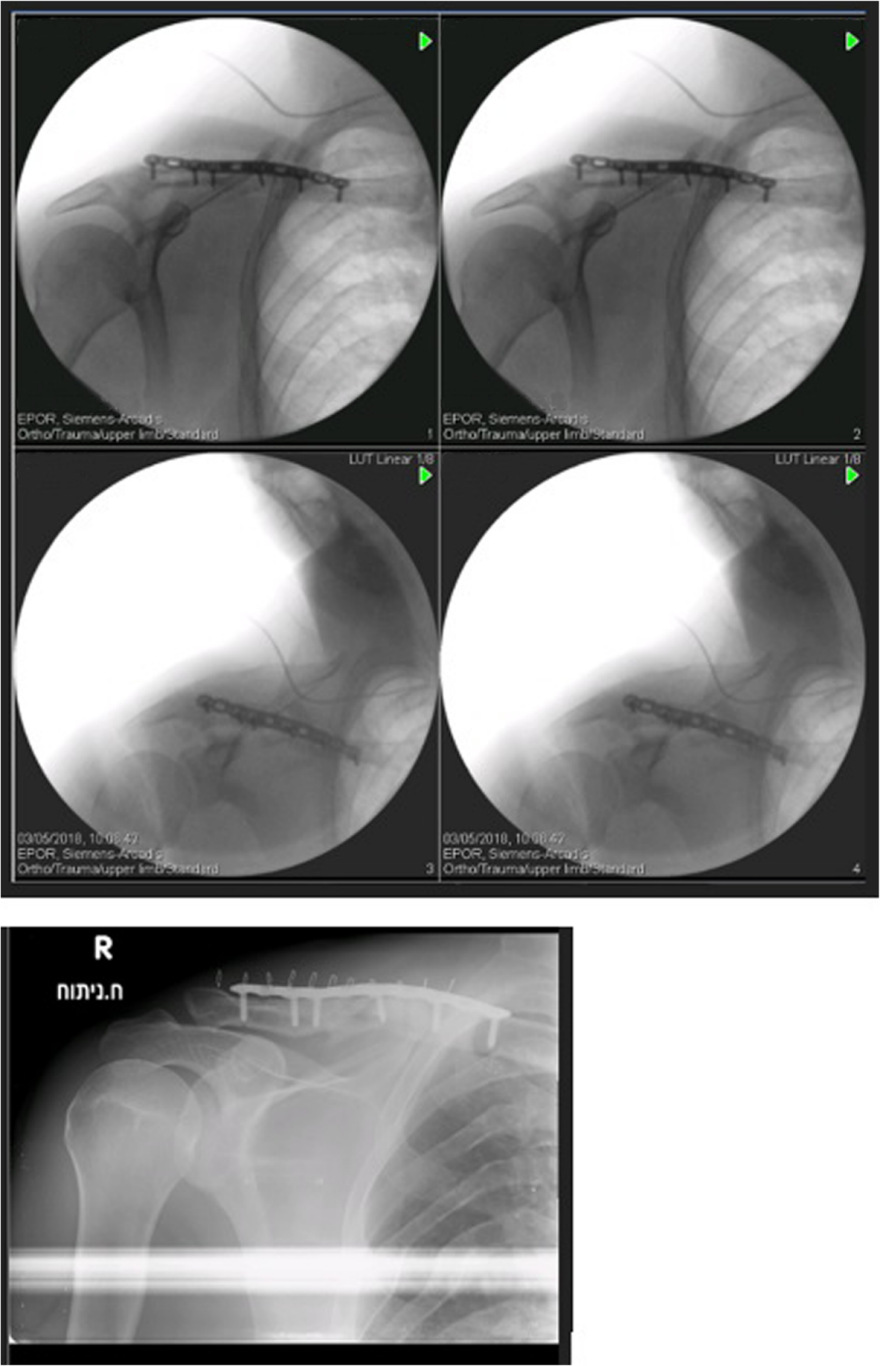
Second operation – intraoperative and postoperative imaging

Five weeks later, he returned to our ER. He described picking up a grocery bag with two packs of sugar, 1 kg each, hearing a breaking sound and feeling his whole shoulder falling down. To our surprise, an X-ray demonstrated a breakage of the fixation clavicle plate with a displacement of the fracture (Fig. 3). He was operated on again: the fracture and implant were exposed, the plate and screws were removed completely, and a new longer fixation plate was implanted (Fig. 4). Furthermore, we used a cancellous bone graft to refill the fracture site. The broken plate was sent back to the factory for inspection.

Our patient gave his consent after he was informed that data concerning his case will be used for research purposes and publication.

## Discussion

The treatment of clavicle fractures is still controversial and debated. Non-operative treatments are the common choice in non-displaced fractures, whereas operative treatments using plates and screws fixation are the current gold standard in displaced and comminuted fractures. The implants that are mostly used can be divided into two groups: intramedullary devices (nails) and extramedullary devices (plates). Plates can be subdivided into reconstruction plates and small fragment locking compression plates. As in any other operative intervention, postoperative complications have been reported. Wijdicks *et al.* published a large systematic review of the complications of plate fixation of clavicle fractures and reported low non-union and malunion rates (< 10%) [12]. Furthermore, they noted that the vast majority of complications seemed to be implant-related, with irritation or failure of the plate consistently reported in almost every study ranging from 9 to 64% of the cases [12]. Failure of the implants is seen in 1 to 4% of the cases [13] and can be related to either a mechanical or a biological mode. Biological reasons include poor bone quality, age, and fracture location. Mechanical reasons include bending stress leading to plate failure usually at the screw–plate junction, screw loosening, and plate breakage [7, 13, 14]. In the former, the mechanism of failure is expressed as a gradual loosening of fixation, leading to pull out of the hardware construct. In the latter, a formal breakage of the hardware occurs, while the screws remain well fixed to the bone without loosening [7]. Some risk factors for plate breakage have been suggested. Among them are high energy injuries, Robinson 2B2 fracture type, using a plate to bridge a fracture, and lifting a heavy weight within 1 month after surgery against rehabilitation program since the plates may not be strong enough to support shoulder motion before bony union [7, 15].

There are different types of bones in the skeleton; the clavicle is classified as a modified long bone whose biomechanical behaviour is unlike a vertical long bone. In vertical long bones gravity applies compression forces along the bone; however, in the clavicle, gravity is perpendicular to the bone due to its horizontal position. In a laboratory environment on 12 fresh cadaveric clavicles, Harnroongroj *et al.* found that the compression load along the axis of the clavicle produced a middle one-third clavicular fracture as in clinical observation [16]. Clavicle anatomy and biomechanics may explain why a bridging plate is a risk factor for plate breakage. Questions about ways to optimize the surgical technique and rehabilitation protocol following clavicle fracture, while considering the characteristics and orientation of the clavicle relative to gravity, should be raised and examined.

In the current case report, our patient was exposed to some of the risk factors for implant failure including the use of a bridging plate and postoperative functional restrictions. Another important aspect, which is less common but is gaining attention, especially among policymakers and health reforms, is patient expectations [17]. We present a scenario where the chain of decisions was in accordance to the patient’s goal (regain function as soon as possible) while considering other factors such as age, level of physical activity, and fracture pattern, yet the outcome was not satisfying. We believe that in our case the risk factor of our patient’s personality, along with the type of fracture, bridging plate, and the nature of forces and stresses acting on the clavicle, led to plate failure and poor surgical outcome. Identifying a patient’s personality (highly motivated to regain functionality) in advance and educating the patient thoroughly on the treatment process including the possible risks and complications that might occur due to poor compliance with the rehabilitation instructions, might have helped in avoiding plate failure.

## Conclusions

This case study presents a rare plate failure expressed as plate breakage. We believe the main reasons for the plate breakage were the type of fracture requiring a bridging plate, the nature of forces and loads on the bone, and lack of setting up clear patient expectations. Future studies should consider the anatomy of the clavicle and think of new ways to accelerate bone growth by applying forces through the bone and not perpendicular to it. Furthermore, it may be assumed that the notion that setting up patient expectations is an important process for good surgical outcome also applies to patients with a displaced midshaft clavicle fracture.

## Abbreviation

ER: Emergency room
